# Molecular Identification and Functional Characterization of LC-PUFA Biosynthesis Elongase (*elovl2*) Gene in Chinese Sturgeon (*Acipenser sinensis*)

**DOI:** 10.3390/ani14162343

**Published:** 2024-08-14

**Authors:** Haoze Ding, Xuetao Shi, Zhengyong Wen, Xin Zhu, Pei Chen, Yacheng Hu, Kan Xiao, Jing Yang, Tian Tian, Dezhi Zhang, Shuqi Wang, Yang Li

**Affiliations:** 1 Hubei Key Laboratory of Three Gorges Project for Conservation of Fishes, Yichang 443100, China; 23hzding@stu.edu.cn (H.D.); shi_xuetao@ctg.com.cn (X.S.); zhu_xin2@ctg.com.cn (X.Z.); chen_pei1@ctg.com.cn (P.C.); hu_yacheng@ctg.com.cn (Y.H.); xiao_kan@ctg.com.cn (K.X.); yang_jing7@ctg.com.cn (J.Y.); tian_tian1@ctg.com.cn (T.T.); zhang_dezhi1@ctg.com.cn (D.Z.); 2 Chinese Sturgeon Research Institute, China Three Gorges Corporation, Yichang 443100, China; 3 Guangdong Provincial Key Laboratory of Marine Biotechnology, Institute of Marine Sciences, Shantou University, Shantou 515063, China; 4 Key Laboratory of Sichuan Province for Fishes Conservation and Utilization in the Upper Reaches of the Yangtze River, Neijiang Normal University, Neijiang 641100, China; zhengyong_wen@126.com

**Keywords:** *elovl2*, functional characterization, LC-PUFAs biosynthesis, Chinese sturgeon (*Acipenser sinensis*), chondrichthyans

## Abstract

**Simple Summary:**

The elongation of very-long-chain fatty acids gene 2 (*elovl2*) of Chinese sturgeon was identified in acipenseriformes species for the first time. Elovl2 was highly conserved in molecular evolution among vertebrates. Functional characterization in yeast demonstrated that Chinese sturgeon Elovl2 could efficiently elongate C_20_ (20:4n-6 and 20:5n-3) and C_22_ (22:4n-6 and 22:5n-3) substrates, confirming its critical roles in long-chain polyunsaturated fatty acid (LC-PUFA) biosynthesis. Diets with different types of PUFAs can affect the transcription levels of *elovl2* in Chinese sturgeon. This study enhances our understanding about the evolutionary and functional characteristics of *elovl2* and also provides novel insights into the mechanism of LC-PUFA biosynthesis in vertebrates.

**Abstract:**

Elongases of very-long-chain fatty acids (Elovls) are critical rate-limiting enzymes that are involved in LC-PUFA biosynthesis through catalyzing the two-carbon elongation of a pre-existing fatty acyl chain. Thus far, several Elovls have been extensively studied in teleost. However, the functional and physiological roles of Elovls in chondrichthyans have rarely been reported. In this study, we identified and characterized *elovl2* from the endangered Chinese sturgeon (*Acipenser sinensis*) by whole genome scanning. The results show that the coding sequence of *elovl2* was 894 bp in length, for a putative protein of 297 amnio acids. Comparative genomic analyses indicated that Chinese sturgeon *elovl2* was evolutionarily conserved. Functional characterization in yeast demonstrated that the Chinese sturgeon Elovl2 could efficiently elongate C_20_ (ARA and EPA) and C_22_ (22:4n-6 and 22:5n-3) substrates, confirming its critical roles in LC-PUFA biosynthesis. Spatial and temporal expression analyses showed high *elovl2* mRNA levels were detected in the liver and brain and showed an increase trend both in embryonic and post-hatching stages. Interestingly, diets with vegetable oils as lipid sources could significantly induce the high expression of *elovl2* in Chinese sturgeon, implying that the endogenous LC-PUFA biosynthesis pathway was stimulated by lack of LC-PUFA in their diets. Our findings will enhance our understanding about the evolutionary and functional roles of *elovl2* and provide novel insights into the LC-PUFA biosynthesis mechanism in vertebrates.

## 1. Introduction

Long-chain polyunsaturated fatty acids (LC-PUFAs), including arachidonic acid (ARA, 20:4n-6), eicosapentaenoic (EPA; 20:5n-3), and docosahexaenoic (DHA; 22:6n-3) acids, have crucial roles in promoting normal growth, development, and reproduction in vertebrates [1]. Due to lack of Δ12 and Δ15 desaturase enzymes, teleost fish are considered to be unable to endogenously synthesize C_18_ PUFAs [2]. However, most teleost fish possess varying capacities to utilize dietary C_18_ PUFAs for the biosynthesis of LC-PUFAs [3,4], while some species completely lack this kind of ability, and, thus, LC-PUFAs are regarded as dietary essential fatty acids for these species [2]. Therefore, it is necessary to elucidate the biosynthesis capacity of LC-PUFAs from C_18_ PUFAs for evaluating the dietary LC-PUFA requirements for normal growth and development in fish species.

**Figure 1:**
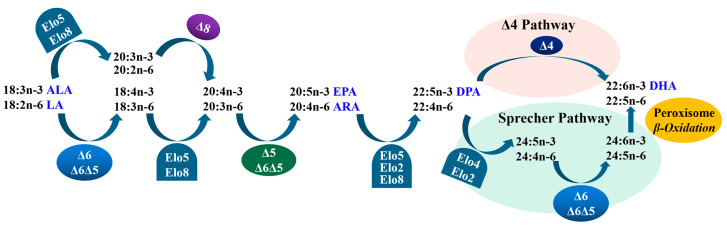
Biosynthetic pathways of LC-PUFA from precursors LA and ALA in teleosts. “∆x” denotes reactions catalyzed by fatty acyl desaturases, Fads; “Elo” indicates reactions catalyzed by elongation of very-long-chain fatty acid proteins, Elovl [5].

In teleosts, LC-PUFAs can generally be biosynthesized with C_18_ PUFAs, such as linoleic acid (LA, 18:2n-6) and α-linolenic acid (ALA, 18:3n-3), through a series of coordinated actions of fatty acyl desaturases (Fads) and Elovl proteins [5] (Figure 1). Fads can introduce a stereospecific double bond by conducting dehydrogenation reactions, while Elovls can elongate the fatty acyl chain by catalyzing the rate-limiting condensation reaction [1,6]. Thus far, four members of Elovls have been identified in teleosts, and they are named as Elovl2, Elovl4, Elovl5, and Elovl8, which generally have an important role in the elongation of LC-PUFAs [4,7].

Zebrafish (*Danio rerio*) Elovl5 was the first elongase to be functionally characterized as a critical enzyme in the elongation step of LC-PUFA biosynthesis [8]. Subsequently, numerous studies have reported the molecular and functional characterizations of Elovl5 from a wide range of fish species [9,10,11,12,13,14,15,16]. These results demonstrated that Elovl5 ubiquitously exists in teleost fish and is primarily involved in elongating C_18_ and C_20_ PUFA substrates [9,12,13,15]. Notably, Elovl5 was also proven to have an elongation capability towards C_22_ PUFAs [14,16]. Similarly, Elovl4 has been extensively investigated in a large variety of fish species, such as zebrafish [8], loach (*Misgurnus anguillicaudatus*) [17], Atlantic salmon (*Salmo salar*) [18], rabbitfish (*Siganus canaliculatus*) [10], cobia (*Rachycentron canadum*) [19], and large yellow croaker (*Pseudosciaena crocea*) [20]. It has been demonstrated that Elovl4 also possessed the capacity to elongate C_18–22_ PUFA substrates [4,5]. 

Unlike Elovl5 and Elovl4, Elovl2 has only been detected in a few fish species, including zebrafish [11], African catfish (*Clarias gariepinus*) [21], rainbow trout (*Oncorhynchus mykiss*) [22], Atlantic salmon [9], European sardine (*Sardina pilchardus*) [23], ambaqui (*Colossoma macropomum*) [24], tench (*Tinca tinca*) [25], sliver barb (*Barbonymus gonionotus*) [26], and Japanese eel (*Anguilla japonica*) [27]. Meanwhile, these studies have revealed that Elovl2 exhibits a preference for elongating C_20_ and C_22_ PUFAs substrates [5]. Interestingly, a novel member of the *elovl* family, named *elovl8*, with two distinct isotypes was identified in rabbitfish [7]. This fish-specific elongase enzyme was demonstrated to exhibit elongation activity towards C_18_ and C_20_ PUFA in both African catfish [21] and rabbitfish [7]. These findings suggested that the evolutionary history and functional characteristics of *elovl* genes are more complex in teleost in comparison with those in mammals.

Chinese sturgeon (*Acipenser sinensis*) is a representative fish in Chondrichthyans, and it is an endangered species that is mainly distributed in the Yangtze River of China [28]. In recent decades, the reproduction of Chinese sturgeon has been seriously impacted due to overfishing and habitat degradation [29]. As a result, this fish has been classified as a critically endangered species by the International Union for Conservation of Nature (IUCN) [30]. Therefore, efficient artificial breeding and releasing technologies play critical roles in preserving Chinese sturgeon. Meanwhile, a better understanding of nutritional requirements is crucial for formulating high-quality diets to facilitate the normal growth and development of fishes [21,31]. However, little is known about the mechanisms underlying LC-PUFA biosynthesis and the dietary essential fatty acid requirements for Chinese sturgeon. Thus, more studies are required to illustrate the potential mechanism of LC-PUFAs biosynthesis in Chinese sturgeon. In this study, an *elovl2* gene was identified in Chinese sturgeon and its functional characterization, spatio-temporal distribution patterns, and transcriptional changes under different nutritional states were clarified. These data will provide potential implications for evaluating the precise requirements of dietary LC-PUFAs for Chinese sturgeon and expand our understanding about LC-PUFA biosynthesis mechanisms in fish.

## 2. Materials and Methods

### 2.1. Experimental Animals and Sample Collection

The feeding trial was conducted at the Chinese Sturgeon Research Institute in Yichang, Central China. Two hundred artificially bred Chinese sturgeon were selected and acclimatized for a period of two weeks. Before the feeding trial, 180 out of 200 healthy, homogeneous-sized fish (with an average initial body weight 55.26 g ± 2.63 g) were selected and randomly divided into six tanks with 30 fish per tank in triplicates per treatment. Throughout the feeding trial, fish were fed with two different experimental diets with either vegetable oil (VO) or fish oil (FO) as lipid sources for a total of eight weeks. The content of these diets was approximately 50.5% crude protein and 9.4% crude lipid, respectively. The compositions of experimental diets were provided in Table S1. During the acclimation period, a total of six fish were randomly chosen for gene molecular cloning and tissue distributional pattern studies. When the experiment finished, six fish were randomly selected from each tank, and then their liver and brain tissues were collected for comparative gene expression analysis using real-time quantitative PCR. Additionally, during the early developmental stages of the Chinese sturgeon, embryos at nine stages and juvenile fish at six stages were sampled for spatio-temporal expression analysis. All collected samples were immediately frozen in liquid nitrogen after dissection and stored at −80 °C for further analyses.

### 2.2. Molecular Cloning of Elovl2 cDNA and qPCR

The genomic DNA sequence of Chinese sturgeon *elovl2* was obtained from our genome database [32] and the cDNA sequence from our transcriptome database (unpublished). Subsequently, gene structure and protein sequence predictions were performed using the online software Softberry: http://www.softberry.com (accessed on 17 June 2023). Additionally, the predicted protein sequence was validated using Standard Protein BLAST (BLASTP) from NCBI. Total RNA extraction from the liver was conducted using Trizol reagent (Invitrogen, Carlsbad, CA, USA), followed by qualitative and quantitative analysis of RNA samples using NanoDrop One Spectrophotometer (Thermo Scientific, Rockford, IL, USA). For reverse transcription of each sample, 1 μg of total RNA was used with FastKing gDNA Dispelling RT SuperMix (Tiangen Biotech, Beijing, China). Two pairs of primers (Table S2) were designed to amplify the full-length coding sequence of Chinese sturgeon *elovl2* using cDNA as a template. PCR amplification procedures and the purification of target products were carried out following methods described in our previous study [7], then cloned into a pCE2-TA/Blunt-Zero vector (Vazyme Biotech, Nanjing, China), and subsequently sequenced at Sangon Biotech. (Shanghai, China).

Quantitative real-time PCR (qPCR) analysis was performed on the ABI 7500 real-time PCR system (Applied Biosystems, Carlsbad, CA, USA) with a total reaction volume of 20 μL, using SuperReal PreMix Plus SYBR Green kit (Tiangen Biotech, Beijing, China). Each reaction mixture contained diluted cDNA along with specific forward and reverse primers (Table S2). All the amplification reactions were conducted in triplicate with the inclusion of non-template control in each reaction. The results of mRNA relative expression levels were normalized by 18S rRNA [33] and calculated using the 2^−ΔΔCT^ method [34]. All the procedures were carried out according to the manufacturers’ protocols.

### 2.3. Bioinformatic Analyses

The validated Chinese sturgeon *elovl2* cDNA sequence was deposited to NCBI (OQ910508) and used for further analyses. The online software AUGUSTUS: https://bioinf.uni-greifswald.de/augustus/ (accessed on 8 September 2023) was applied to predict the open reading frames (ORFs) and encoded protein sequence of Chinese sturgeon *elovl2*. Transmembrane domains of Chinese sturgeon putative Elovl2 protein were identified by DeepTMHMM [35]. The conserved motifs were identified according to previous studies [9,27]. Subsequently, multiple sequence alignment of Elovl2s protein sequences from six representative vertebrate species including Chinese sturgeon was performed using ClustalW 2.0 [36] and BioEdit 7.0 software [37]. In addition, 3D structures of Elovl2s from six vertebrates were obtained using AlphaFold3 online server [38]. Subsequently, the models with the highest-ranking scores were selected and modified by PyMol 2.5 for visualization of important regions [39]. Furthermore, a comparative genomic survey was conducted to recognize genetic loci and gene structures of Chinese sturgeon *elovl2*. The PDB files and confidence evaluations of the Elovl2s protein models we used are available in the Supplementary files.

To investigate the phylogenetic relationship of the *elovl* genes among vertebrates, a phylogenetic analysis was conducted based on Elovl protein sequences downloaded from NCBI or the Ensembl database. Multiple protein sequence alignment was performed using ClustalW 2.0, as previously described. Subsequently, the aligned datasets were used to construct a phylogenetic tree by the Neighbor Joining (NJ) approach in MEGA 11.0 software [40]. The JTT + G model was selected as the best model after the evaluation of the Bayesian Information Criterion (BIC) index. Confidence in the branch topology of the resulting phylogenetic tree was assessed through bootstrapping with 1000 resampling replicates and the resulting tree was rooted with *elovl6* sequences. The accession numbers for selected Elovl proteins are listed in Table S3.

### 2.4. Functional Characterization in Yeast 

The cDNA synthesized from Chinese sturgeon liver tissue was used as a template to amplify the PCR fragments corresponding to the coding sequence region of Chinese sturgeon *elovl2*, using 2 × Phanta Max Master Mix (Vazyme Biotech, Nanjing, China). Primers containing KpnI restriction enzyme site (forward) and EcoRI restriction enzyme site (reverse), as listed in Table S2, were used in a PCR reaction with an initial denaturation at 95 °C for 3 min, followed by 30 cycles of denaturation at 95 °C for 15 s, annealing at 56 °C for 15 s, extension at 72 °C for 90 s, and a final extension step at 72 °C for 5 min. Subsequently, the DNA fragments were purified by a FastPure Gel DNA Extraction Mini Kit (Vazyme Biotech, Nanjing, China) and were then digested with the corresponding restriction endonucleases (Takara Biotech, Dalian, China) and ligated into a similarly restricted yeast expression vector pYES2 (Invitrogen, Carlsbad, CA, USA). The purified constructed recombinant plasmid was used to transform *Saccharomyces cerevisiae* competent cells following the S.c EasyComp Transformation Kit protocol (Invitrogen, Carlsbad, CA, USA). The transformation and selection of yeast with recombinant plasmid (pYES2_*elovl2*) and yeast culture were performed according to the methods described by previous studies [41]. Recombinant yeast was incubated with one of the potential fatty acid substrates, including 18:2n-6, 18:3n-3, 18:3n-6, 18:4n-3, 20:4n-6, 20:5n-3, 22:4n-6, and 22:5n-3 (Cayman Chemical Company Inc., Ann Arbor, MI, USA), which were added at final concentrations of 0.5 mM (C_18_), 0.75 mM (C_20_), and 1.0 mM (C_22_). After two days of incubation, the yeast cells were harvested and washed as previously described [42].

### 2.5. Fatty Acid Analysis

The total lipids of yeast samples were extracted using chloroform/methanol (2:1, *v*/*v*) containing 0.01% BHT as an antioxidant, following the method described by [17]. Fatty acid methyl esters (FAMEs) were prepared and purified according to the procedure described by Christie [43]. Identification and quantification of FAMEs were performed using gas chromatography mass spectrometry (GC-2010 Pro, Shimadzu, Kyoto, Japan). The conversion of PUFA substrate was calculated based on the proportion of fatty acid (FA) substrate converted into elongated FA products, represented as [individual product area/(all product areas + substrate area)] × 100.

### 2.6. Statistical Analysis

The statistical analyses were conducted using SPSS 19.0 (IBM, Armonk, NY, USA) and GraphPad Prism 6.0 (San Diego, CA, USA). All the data were presented as means ± standard error of the mean (SEM). One-way analysis of variance (ANOVA) was used to analyze qPCR expression data, followed by Tukey’s multiple comparison test or Student’s *t*-test, implemented in the Origin 7.0 software. Significance was determined at *p* < 0.05.

## 3. Results

### 3.1. Molecular Identification of Chinese Sturgeon Elovl2 

**Figure 2:**
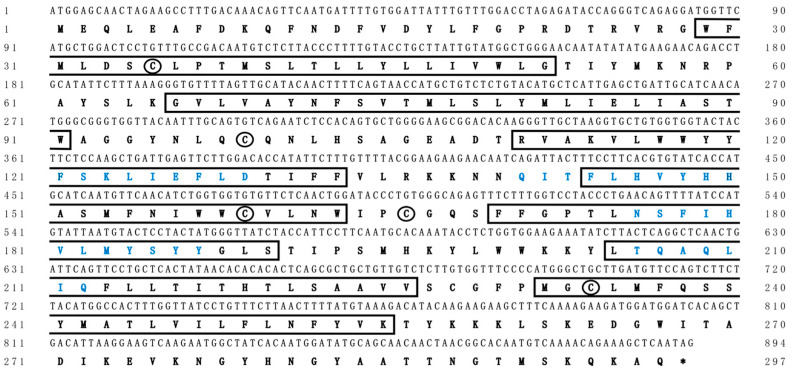
Complete coding sequences and deduced protein sequences of the *elovl2* cDNA of Chinese sturgeon. Positions of nucleotides and amino acids are numbered on each side. Conserved transmembrane domains are boxed. Amino acids sequences in blue type represent the conserved motifs of fatty acid elongases. Cysteines in the two proteins are indicated by circles. Asterisks (*) represents the stop codon.

In this study, we identified the cDNA sequence by integrating genome scanning, bioinformatic analyses, and molecular experiments. The ORF of Chinese sturgeon *elovl2* was 894 bp in length, which was predicted to encode a protein with 297 putative amino acid residues (Figure 2). The deduced protein was recognized as a membrane protein and it contained seven transmembrane hydrophobic α-helices and four conserved motifs of elongases (Figure 2). Meanwhile, five cysteine residues, important amino acids for maintaining the tertiary structure of proteins, were identified in this elongase protein. The cDNA sequence of Chinese sturgeon *elovl2* was deposited in the GenBank database under an accession number OQ910508.

### 3.2. Multiple Protein Sequence Alignments of the Elovl2 among Vertebrates

**Figure 3:**
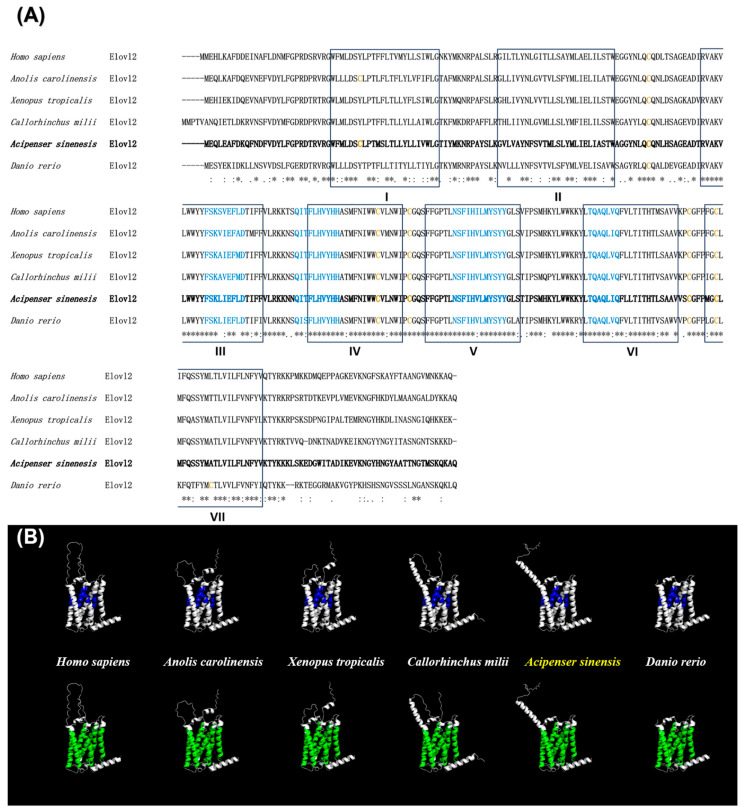
Multiple protein sequences alignment (**A**) and 3D structural prediction (**B**) of Elovl2s between Chinese sturgeon and five other vertebrates. Seven putative transmembrane domains are boxed and labeled with I to VII, respectively. Four conserved motifs of elongase are marked in blue type. Cysteines are marked in brown type. Asterisks (*) indicate the conservation of the amino acids among these sequences (**A**). The transmembrane regions and conserved motifs are colored in blue and green in the 3D structure, respectively (**B**).

Multiple protein sequence alignments can enhance our understanding of the structural characteristics of the examined proteins. In this study, multiple Elovl2 proteins among vertebrates were performed to reveal the structural characteristics of this kind of elongase. The results shows that Elovl2 was highly conserved among vertebrates, containing seven transmembrane domains, four conserved elongase motifs, and five highly conserved cysteine residues (Figure 3A). Moreover, the Chinese sturgeon Elovl2 shares a relatively higher sequence identity with that in other selected vertebrate species including human (*Homo sapiens*, 73.51%), green anole (*Anolis carolinensis*, 75.17%), common frog (*Rana temporaria*, 75.17%), elephant shark (*Callorhinchus milii*, 73.18%), and zebrafish (70.2%) (Table S4). The 3D structure predictions revealed that the functional motifs and transmembrane regions of Elovl2s are conserved among vertebrates (Figure 3B).

### 3.3. Synteny and Gene Structure Comparison of the Elvol2 in Vertebrates

**Figure 4:**
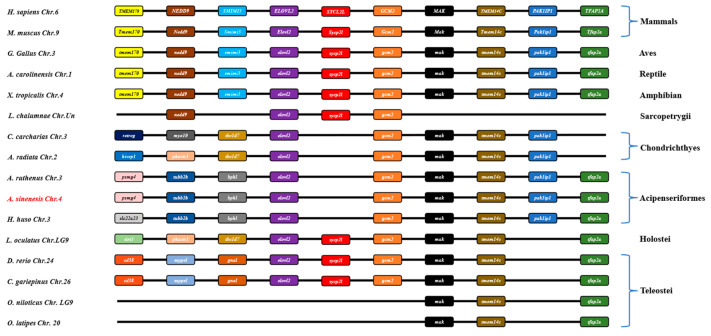
Synteny comparisons of *elovl2s* in vertebrates. The colored blocks represent different genes. The solid lines represent intergenic regions. The target species of the present study is marked in red type.

In this study, a comparative genomic synteny analysis was conducted across various vertebrate species to further reveal the genetic and evolutionary characteristics in vertebrate. As shown in Figure 4, *elovl2* was widely identified in vertebrate genomes and multiple genes downstream of *elovl2* exhibited similar a gene order and position in vertebrates, including mammals, aves, reptiles, amphibians, sarcopterygii, chondrichthyes, acipenseriformes, holostei, and some teleostei species like zebrafish and African catfish (Figure 4). Notably, *elovl2* possessed a similar gene order and genetic location in acipenseriformes species, including sterlet (*Acipenser ruthenus*), Chinese sturgeon, and beluga (*Huso huso*) (Figure 4). Consistently, a gene cluster *elovl2-gcm2-mak-tmem14c-pak1ip1-tfaap2a* was identified within these fish genomes (Figure 4). Additionally, the gene cluster *elovl2-gcm2-mak-tmem14c-pak1ip1* was also identified in chondrichthyes species, including great white shark (*Carcharodon carcharias*) and thorny skate (*Amblyraja radiata*) (Figure 4). 

**Figure 5:**
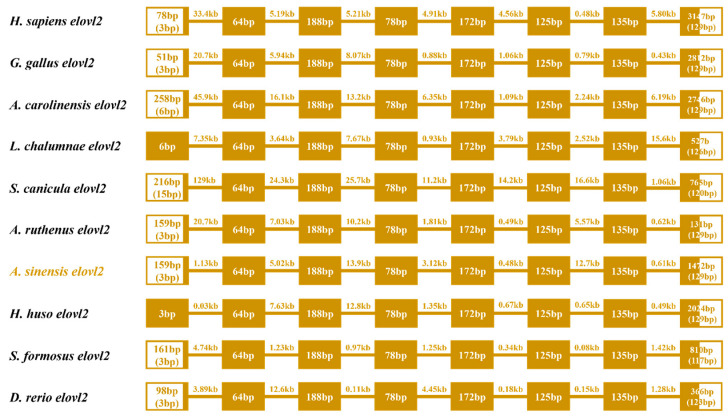
Comparative analysis of the gene structures of *elovl2s* among different species. The colored boxes and lines represent the exons and introns, respectively. Boxes in blank indicate the untranslated regions. Numbers in boxes and on lines represent the length of exons and introns, respectively.

Gene structure analysis showed that eight exons and seven introns were observed in the ten representative vertebrate species, sharing a similar gene structure (Figure 5). However, some variations were found both in the first and eighth exons, while the other exons were highly conserved in length. Meanwhile, the length of introns was also variable in these selected species (Figure 5). 

### 3.4. Phylogenic Analysis

**Figure 6:**
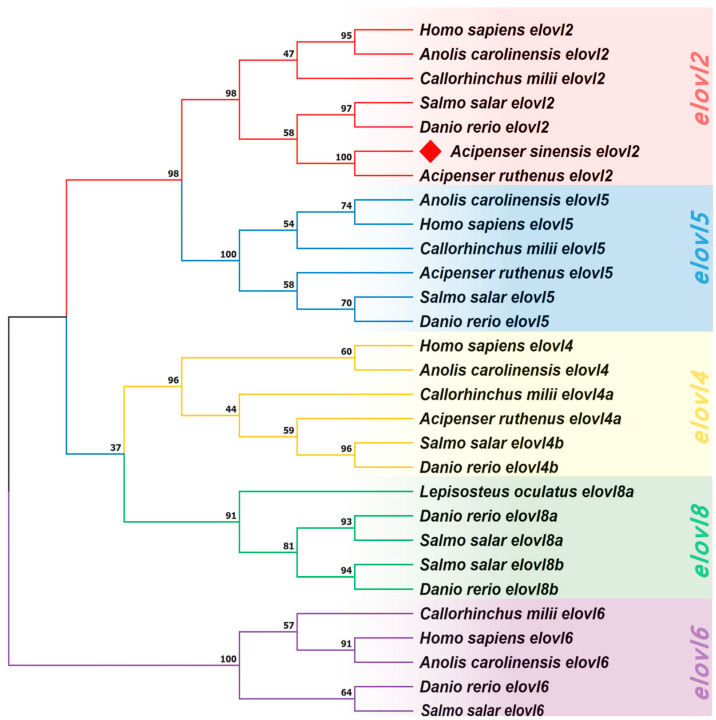
A phylogenetic tree inferring the relationships of vertebrate *elovl* genes. The tree was constructed by the Neighbor Joining (NJ) method based on a dataset of amino acids. Values at the nodes represent bootstrap percentages from 1000 replicates. The target species is marked by a diamond. The tree is rooted on the elovl6 clade.

A Neighbor Joining (NJ) tree was generated to help better understanding the evolutionary relationships of Elovl2s among vertebrates (Figure 6). The results show that the phylogenetic tree was divided into five clusters including *elovl2*, *elovl5*, *elovl4*, *elovl8*, and *elovl6* subfamilies (Figure 6), and the *elovl2* subfamily was adjacent to the *elovl5* subfamily (Figure 6). Furthermore, the Chinese sturgeon putative *elovl2* was clustered in the *elovl2* subfamily and shared its closest relationship with sterlet (*Acipenser ruthenus*) *elovl2* (Figure 6). The phylogenetic tree had high node support rates. 

### 3.5. Spatial and Temporal Distribution Patterns of Chinese Sturgeon Elovl2 

**Figure 7:**
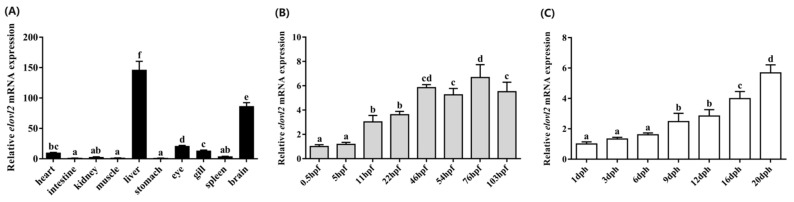
Relative mRNA spatial (**A**) and temporal (**B**,**C**) distribution patterns of the Chinese sturgeon’s *elovl2*. Ten tissues including the heart, intestine, kidney, muscle, liver, stomach, eye, gill, spleen, and brain were analyzed (**A**). Eight stages embryos (0.5, 5, 11, 22, 46, 54, 76, and 103 h post-fertilization) (**B**) and seven stages fish (1, 3, 6, 9, 12, 16, and 20 days post-hatching) were analyzed (**C**). Data are means ± SEM (*n* = 6). Different lowercase letters above the bars denotes significant differences among the treatments (*p* < 0.05).

Spatial and temporal distribution patterns of *elovl2* in Chinese sturgeon were determined by qPCR. In brief, a total of 10 tissues including the brain, spleen, gill, eye, stomach, liver, muscle, kidney, intestine, and heart were analyzed, and the results show that *elovl2* was widely expressed in all of the detected tissues, with significantly higher expression levels in the liver and brain compared to other tissues (Figure 7). The relative expression levels were ranked as follows: liver > brain > eye > gill > heart > spleen > kidney > muscle > intestine > stomach (Figure 7A). Additionally, embryos at eight different stages (0.5, 5, 11, 22, 46, 54, 76, and 103 h post-fertilization) and fingerlings at seven different stages (1, 3, 6, 9, 12, 16, and 20 days post-hatching), representing various developmental stages of the Chinese sturgeon, were examined. The temporal distribution pattern of *elovl2* mRNA expression levels exhibited a progressive increase throughout both embryo and post-hatching stages (Figure 7B,C). During the embryonic stages, the lowest expression level of Chinese sturgeon *elovl2* was detected at 0.5 hpf. The expression level of *elovl2* showed an increasing trend from 0.5 to 46 hpf. The transcriptional profile of *elovl2* in Chinese sturgeon during the post-hatching stages showed significantly higher expression levels at the exogenous nutrition stages (16 and 20 dph) in comparison with the endogenous nutrition stage (1, 3, 6, 9, and 12 dph, Figure 7C).

### 3.6. Functional Characterization of Chinese Sturgeon Elovl2

Potential functions of the putative Elovl2 elongase in Chinese sturgeon were evaluated through heterologous expression of *elovl2* in the yeast *S. cerevisiae*. The yeast was cultured in medium supplemented with one of the following fatty acid substrates: 18:2n-6, 18:3n-3, 18:3n-6, 18:4n-3, 20:4n-6, 20:5n-3, 22:4n-6, or 22:5n-3. The fatty acid composition analysis of yeast transformed with the empty vector (pYES2) revealed a deficiency in PUFAs elongase activity in the recombinant yeast. In yeast transformed with the Chinese sturgeon Elovl2, an additional peak was detected with the added 20:4n-6, 20:5n-3, 22:4n-6, and 22:5n-3 substrates, respectively. GC-MS analyses revealed that Chinese sturgeon Elovl2 had the capacity to elongate C_20_ and C_22_ LC-PUFAs with different conversion rates: 6.99% for 20:4n-6 to 22:4n-6, 12.58% for 20:5n-3 to 22:5n-3, 17.48% for 22:4n-6 to 24:4n-6, and 29.28% for 22:5n-3 to 24:5n-3 (Table 1).

### 3.7. Effect of Dietary Lipid Sources on the Elovl2 Expression in Chinese Sturgeon

**Figure 8:**
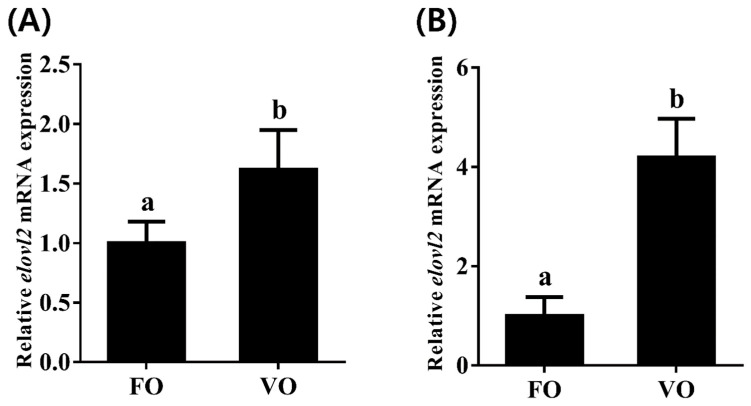
The expressions of *elovl2* mRNA in different tissues ((**A**), liver; (**B**), brain) of Chinese sturgeon fed diets containing fish oil (FO) or vegetable oil (VO). Data are means ± SEM (n = 6). Different lowercase letters above the bars denote significant differences between diets (*p* < 0.05).

To investigate transcriptional change patterns of Chinese sturgeon *elovl2* in response to different dietary lipid sources, we detected the transcription levels of *elovl2* in both liver and brain tissues. We observed that the hepatic mRNA expression of *elovl2* was significantly higher in fish fed the VO diet than that of those fed the FO diet (Figure 8A). Similarly, a consistent result was observed in brain tissue, showing that the VO diet but not the FO diet significantly induced the high transcription levels of *elovl2* in Chinese sturgeon (Figure 8B).

## 4. Discussion

Elovl2 is a critical rate-limiting enzyme involved in the biosynthesis of LC-PUFAs in both mammals and teleosts [4]. In this study, we identified *elovl2* in the Chinese sturgeon for the first time. The coding sequence of Chinese sturgeon *elovl2* was 894 bp and was predicted to encode a protein consisting of 297 amino acids. Meanwhile, multiple protein sequence alignments and 3D structural predictions revealed that Chinese sturgeon Elovl2 possessed typical elongase features similar to homologues from other vertebrate species, including the conserved transmembrane domains and the histidine box (HXXHH) [9,11,27], implying that Elovl2 may play similar roles in LC-PUFAs biosynthesis. Furthermore, the Chinese sturgeon Elovl2 protein shared a relatively higher identity (70–76%) with other vertebrate species including human, green anole, common frog, elephant shark, and zebrafish, which further confirms the consistent roles of Elovl2 in vertebrates. Taken together, these findings suggested that Chinese sturgeon *elovl2* might encode an elongase involved in LC-PUFA biosynthesis.

Comparative genetic synteny showed that *elovl2* is widely present throughout vertebrates, including aves, reptiles, amphibians, sarcopterygii, chondrichthyes, acipenseriformes, and holostei and similar results can also be observed in several studies [4], indicating this gene was evolutionary conserved. Interestingly, we found that *elovl2* was retained in several teleost fish such as zebrafish and African catfish, while it had been lost in Nile tilapia and Japanese medaka. Similarly, previous studies have also indicated that *elovl2* was lost in the Neoteleostei and has only been reported in a few teleost species [24,44]. These results might be associated with the teleost-specific whole genome duplication event, which is considered an important force that drives the biological evolution of fish [45,46,47]. Additionally, the common gene orders and the genetic loci of *elovl2* were found in acipenseriformes species, which shared the same gene cluster *elovl2-gcm2-mak-tmem14c-paklip-tfap2a*. These results confirm the existence of *elovl2* gene in these ancient lineages, similar to findings in elephant shark (*Callorhinchus milii*) [44] and Japanese eel [27]. Furthermore, the gene structure of *elovl2* were also highly conserved, consisting of an identical number of exons and introns, implying that the physiological functions of Elovl2 could be conservative throughout the evolutionary process. Phylogenetic analysis indicates that the *elovl2* clade was clearly different from other elongases, separated from the other clades of *elovl4*, *elovl5*, *elovl8,* and *elovl6*. Additionally, we found that the *elovl2* subgroup was more closely related to the *elovl5* cluster, implying that *elovl2* shared a close relationship with *elovl5*, and, thus, their functional traits may also be similar. These results are consistent with a comprehensive evolutionary study on *elovls* in chordates, which demonstrates that the diversification of *elovl2* and *elovl5*, which are involved in LC-PUFA biosynthesis in vertebrates, expanded from the *elovl 2/5* in chordate ancestry [4]. However, the detailed mechanisms that lead to the diversification of *elovl2* and *elovl5* are still rarely known, but it is reasonable to speculate that Elovl2 and Elovl5 may exert similar physiological functions in the biosynthesis of LC-PUFAs.

The spatial and temporal distribution patterns of *elovl2* in Chinese sturgeon were determined using qPCR. We found that this gene was distributed in all the tissues of Chinese sturgeon that were analyzed, with high expression levels in the liver and brain, suggesting that this gene might play an important role in LC-PUFAs biosynthesis as these tissues are major metabolic sites for the biosynthesis of LC-PUFAs in fish [7,48,49,50]. It is well known that LC-PUFAs, such as EPA and DHA, are necessary for extensive organogenesis and tissue remodeling, particularly crucial for the proper development of the neural and visual systems during embryonic and larval stages [2,51,52]. In this study, we found that the *elovl2* mRNA expression level was consistently increased along with the development of Chinese sturgeon embryos and larvae. Similarly, the transcription levels of *elovl2* had been detected throughout embryonic development in zebrafish and with evident increased expression during the late embryonic development stages in this fish [11]. These findings suggest that the transcription level of *elovl2* was initiated to satisfy the high requirement for LC-PUFAs in embryogenesis and larval development stages in Chinese sturgeon [53,54].

Functional characterization in yeasts demonstrated that Chinese sturgeon Elovl2 has the ability to efficiently elongate C_20_ (ARA and EPA) and C_22_ (22:4n-6 and 22:5n-3) PUFAs to longer-chain PUFAs, and similar results can be observed in rainbow trout [22] and sliver barb [26], indicating Elovl2 should be involved in the process of LC-PUFAs biosynthesis in these fish. Interestingly, activity towards C_18_ PUFA substrates was observed in orthologs of Elovl2 from other fish species including zebrafish [11], African catfish [44], Atlantic salmon [9], tench [25], tambaqui [24], and Japanese eel [27]. It is unclear what caused the difference in the capability in elongated C_18_ PUFA of Elovl2 among fish species, which might be relational to this enzyme’s shared evolutionary origin with Elovl5 [4]. However, there is no doubt that both C_20_ and C_22_ PUFAs are more appropriate elongation substrates for Elovl2. Generally, the substrate preference of enzymes involved in LC-PUFA biosynthesis might reflect the different requirements for physiological functions of n-3 and n-6 LC-PUFAs in fish [55]. In this study, we observed that the conversion rates of Chinese sturgeon Elovl2 towards n-3 PUFA (EPA, 12.6% and 22:5n-3, 29.3%) substrates were generally higher than those towards n-6 PUFA (ARA, 7.0% and 22:4n-6, 17.48%) substrates. The substrate preference of Chinese sturgeon Elovl2 for EPA and 22:5n-3 suggested a strong capacity for DHA biosynthesis in this kind of fish, because the ability of fish to elongate 22:5n-3 to 24:5n-3 is considered crucial for the biosynthesis of DHA through the Sprecher pathway [4,12,27,56].

Previous studies have indicated that dietary nutrition affects the expression of genes involved in LC-PUFA biosynthesis [20,57]. Therefore, the nutritional regulation of these genes has been widely studied in fish [7,13,58,59]. However, related studies on fish *elovl2* have rarely been reported, except in Atlantic salmon and silver barb [9,26,56,60]. It is well known that FO is rich in n-3 LC-PUFAs, such as EPA and DHA, while VO is devoid of these FAs but generally rich in C_18_ PUFAs, LA, and ALA [61,62,63]. In Atlantic salmon, the expression of *elovl2* in liver and intestine was significantly higher in fish fed with VO than in those fed with FO diet [9,60]. In silver barb, diets with mixed VO (50% linseed and 50% corn oil) increased the expression of *elovl2* in liver, intestine, and brain tissues [26]. Consistently, we found that the expression levels of *elovl2* in the liver and brain of Chinese sturgeon fed with VO diets were significantly higher than in those fed with FO diets, indicating that Chinese sturgeon Elovl2 could endogenously biosynthesize LC-PUFA by using dietary C_18_ PUFAs as precursors. As an important elongase involved in LC-PUFA biosynthesis, the higher transcription of Chinese sturgeon *elovl2* in the VO group might represent a physiological adaptation to dietary LC-PUFA deficiencies by increasing LC-PUFA biosynthesis.

## 5. Conclusions

In this study, we identified *elovl2* in Chinese sturgeon and its functional roles involved in LC-PUFA biosynthesis were characterized for the first time. Our results indicated that the Chinese sturgeon Elovl2 enzyme could efficiently elongate C_20_ (ARA and EPA) and C_22_ (22:4n-6 and 22:5n-3) substrates, confirming its potential roles in LC-PUFA biosynthesis. We also investigated the spatio-temporal distribution patterns of Chinese sturgeon *elovl2* and its expression patterns in response to different dietary lipid sources, which further revealed its roles in LC-PUFAs biosynthesis. Our findings will improve our understanding about the evolutionary and functional characteristics of *elovl2* in vertebrates. In the future, we will continue to study other critical enzymes involved in LC-PUFA biosynthesis in Chinese sturgeon and investigate their potential regulation mechanisms. The expected results will provide an important breakthrough in the fundamental understanding of fish LC-PUFA metabolism as well as a theoretical basis for oil source selectivity in formula diets and essential fatty acids requirement for Chinese sturgeon, which could eventually promote the development of the Chinese sturgeon fry industry.

## Figures and Tables

**Table 1:** Functional characterization of Chinese sturgeon Elovl2 elongase: conversions on polyunsaturated fatty acid (FA) substrates.

Substrate	Product	Elovl2	Activity
18:2n-6	20:2n-6	0	C18 → 20
18:3n-3	20:3n-3	0	C18 → 20
18:3n-6	20:3n-6	0	C18 → 20
18:4n-3	20:4n-3	0	C18 → 20
20:4n-6	22:4n-6	6.99	C20 → 22
20:5n-3	22:5n-3	12.58	C20 → 22
22:4n-6	24:4n-6	17.48	C22 → 24
22:5n-3	24:5n-3	29.28	C22 → 24

Results are expressed as percentage of total FA substrate converted to elongated product.

## Data Availability

The data presented in this study are available on request from the corresponding author.

## Supplementary Materials

The following supporting information can be downloaded at: https://www.mdpi.com/article/10.3390/ani14162343/s1, Table S1: Formulation and proximate composition of the experimental diets.; Table S2: Nucleotide sequences of the primers used for PCR; Table S3: *elovl* IDs used for phylogenetic analysis. Data were collected from Ensembl or GenBank; Table S4: Sequence identity calculation of Elovl2s among different species.

## References

[1] Wen Z., Li Y., Bian C., Shi Q., Li Y. (2020). Genome-wide identification of a novel *elovl4* gene and its transcription in response to nutritional and osmotic regulations in rabbitfish (*Siganus canaliculatus*). Aquaculture.

[2] Tocher D.R. (2010). Fatty acid requirements in ontogeny of marine and freshwater fish. Aquac. Res..

[3] Pereira S.L., Leonard A.E., Mukerji P. (2003). Recent advances in the study of fatty acid desaturases from animals and lower eukaryotes. Prostaglandins Leukot. Essent. Fat. Acids.

[4] Castro L.F.C., Tocher D.R., Monroig O. (2016). Long-chain polyunsaturated fatty acid biosynthesis in chordates: Insights into the evolution of *Fads* and *Elovl* gene repertoire. Prog. Lipid Res..

[5] Xie D., Chen C., Dong Y., You C., Wang S., Monroig Ó., Tocher D.R., Li Y. (2021). Regulation of long-chain polyunsaturated fatty acid biosynthesis in teleost fish. Prog. Lipid Res..

[6] Nugteren D.H. (1965). The enzymic chain elongation of fatty acids by rat-liver microsomes. Biochim. Biophys. Acta.

[7] Li Y., Wen Z., You C., Xie Z., Tocher D.R., Zhang Y., Wang S., Li Y. (2020). Genome wide identification and functional characterization of two LC-PUFA biosynthesis elongase (*elovl8*) genes in rabbitfish (*Siganus canaliculatus*). Aquaculture.

[8] Agaba M., Tocher D.R., Dickson C.A., Dick J.R., Teale A.J. (2004). Zebrafish cDNA encoding multifunctional Fatty Acid elongase involved in production of eicosapentaenoic (20:5n-3) and docosahexaenoic (22:6n-3) acids. Mar. Biotechnol..

[9] Morais S., Monroig O., Zheng X., Leaver M.J., Tocher D.R. (2009). Highly Unsaturated Fatty Acid Synthesis in Atlantic Salmon: Characterization of *ELOVL5*- and *ELOVL2*-like Elongases. Mar. Biotechnol..

[10] Monroig Ó., Wang S., Zhang L., You C., Tocher D.R., Li Y. (2012). Elongation of long-chain fatty acids in rabbitfish (*Siganus canaliculatus*): Cloning, functional characterisation and tissue distribution of *Elovl5*- and *Elovl4*-like elongases. Aquaculture.

[11] Monroig Ó., Tocher D.R., Hontoria F., Navarro J.C. (2013). Functional characterisation of a *Fads2* fatty acyl desaturase with Δ6/Δ8 activity and an *Elovl5* with C16, C18 and C20 elongase activity in the anadromous teleost meagre (*Argyrosomus regius*). Aquaculture.

[12] Wang S., Monroig Ó., Tang G., Zhang L., You C., Tocher D.R., Li Y. (2014). Investigating long-chain polyunsaturated fatty acid biosynthesis in teleost fish: Functional characterization of fatty acyl desaturase (*Fads2*) and *Elovl5* elongase in the catadromous species, Japanese eel (*Anguilla japonica*). Aquaculture.

[13] Kuah M.-K., Jaya-Ram A., Shu-Chien A.C. (2015). The capacity for long-chain polyunsaturated fatty acid synthesis in a carnivorous vertebrate: Functional characterisation and nutritional regulation of a *Fads2* fatty acyl desaturase with Δ4 activity and an *Elovl5* elongase in striped snakehead (*Channa striata*). Biochim. Biophys. Acta.

[14] Xie D., Chen F., Lin S., You C., Wang S., Zhang Q., Monroig Ó., Tocher D.R., Li Y. (2016). Long-chain polyunsaturated fatty acid biosynthesis in the euryhaline herbivorous teleost *Scatophagus argus*: Functional characterization, tissue expression and nutritional regulation of two fatty acyl elongases. Comp. Biochem. Physiol. B Biochem. Mol. Biol..

[15] Zou W., Lin Z., Huang Y., Limbu S.M., Wen X. (2019). Molecular cloning and functional characterization of elongase (*elovl5*) and fatty acyl desaturase (*fads2*) in sciaenid, *Nibea diacanthus* (Lacepède, 1802). Gene.

[16] Galindo A., Garrido D., Monroig Ó., Pérez J.A., Betancor M.B., Acosta N.G., Kabeya N., Marrero M.A., Bolaños A., Rodríguez C. (2021). Polyunsaturated fatty acid metabolism in three fish species with different trophic level. Aquaculture.

[17] Yan J., Liang X., Cui Y., Cao X., Gao J. (2018). *Elovl4* can effectively elongate C18 polyunsaturated fatty acids in loach *Misgurnus anguillicaudatus*. Biochem. Biophys. Res. Commun..

[18] Carmona-Antoñanzas G., Monroig O., Dick J.R., Davie A., Tocher D.R. (2011). Biosynthesis of very long-chain fatty acids (C>24) in Atlantic salmon: Cloning, functional characterisation, and tissue distribution of an *Elovl4* elongase. Comp. Biochem. Physiol. B Biochem. Mol. Biol..

[19] Monroig Ó., Webb K., Ibarra-Castro L., Holt G.J., Tocher D.R. (2011). Biosynthesis of long-chain polyunsaturated fatty acids in marine fish: Characterization of an *Elovl4*-like elongase from cobia *Rachycentron canadum* and activation of the pathway during early life stages. Aquaculture.

[20] Li S., Monroig Ó., Wang T., Yuan Y., Carlos Navarro J., Hontoria F., Liao K., Tocher D.R., Mai K., Xu W. (2017). Functional characterization and differential nutritional regulation of putative *Elovl5* and *Elovl4* elongases in large yellow croaker (*Larimichthys crocea*). Sci. Rep..

[21] Oboh A., Betancor M.B., Tocher D.R., Monroig O. (2016). Biosynthesis of long-chain polyunsaturated fatty acids in the African catfish *Clarias gariepinus*: Molecular cloning and functional characterisation of fatty acyl desaturase (*fads2*) and elongase (*elovl2*) cDNAs. Aquaculture.

[22] Gregory M.K., James M.J. (2014). Rainbow trout (*Oncorhynchus mykiss*) *Elovl5* and *Elovl2* differ in selectivity for elongation of omega-3 docosapentaenoic acid. Biochim. Biophys. Acta.

[23] Machado A.M., Tørresen O.K., Kabeya N., Couto A., Petersen B., Felício M., Campos P.F., Fonseca E., Bandarra N., Lopes-Marques M. (2018). “Out of the Can”: A Draft Genome Assembly, Liver Transcriptome, and Nutrigenomics of the European Sardine, *Sardina pilchardus*. Genes.

[24] Ferraz R.B., Kabeya N., Lopes-Marques M., Machado A.M., Ribeiro R.A., Salaro A.L., Ozório R., Castro L.F.C., Monroig Ó. (2019). A complete enzymatic capacity for long-chain polyunsaturated fatty acid biosynthesis is present in the Amazonian teleost tambaqui, *Colossoma macropomum*. Comp. Biochem. Physiol. B Biochem. Mol. Biol..

[25] Garrido D., Monroig Ó., Galindo A., Betancor M.B., Pérez J.A., Kabeya N., Marrero M., Rodríguez C. (2020). Lipid metabolism in *Tinca tinca* and its n-3 LC-PUFA biosynthesis capacity. Aquaculture.

[26] Janaranjani M., Shu-Chien A.C. (2020). Complete repertoire of long-chain polyunsaturated fatty acids biosynthesis enzymes in a cyprinid, silver barb (*Barbonymus gonionotus*): Cloning, functional characterization and dietary regulation of *Elovl2* and *Elovl4*. Aquac. Nutr..

[27] Xu W., Wang S., You C., Zhang Y., Monroig Ó., Tocher D.R., Li Y. (2020). The catadromous teleost *Anguilla japonica* has a complete enzymatic repertoire for the biosynthesis of docosahexaenoic acid from α-linolenic acid: Cloning and functional characterization of an *Elovl2* elongase. Comp. Biochem. Physiol. B Biochem. Mol. Biol..

[28] Peng Z., Ludwig A., Wang D., Diogo R., Wei Q., He S. (2007). Age and biogeography of major clades in sturgeons and paddlefishes (Pisces: *Acipenseriformes*). Mol. Phylogenet. Evol..

[29] Zhuang P., Zhao F., Zhang T., Chen Y., Liu J., Zhang L., Kynard B. (2016). New evidence may support the persistence and adaptability of the near-extinct Chinese sturgeon. Biol. Conserv..

[30] Huang Z., Wang L. (2018). Yangtze Dams Increasingly Threaten the Survival of the Chinese Sturgeon. Curr. Biol..

[31] Zhang Y., Lu R., Qin C., Nie G. (2020). Precision nutritional regulation and aquaculture. Aquac. Rep..

[32] Wang B., Wu B., Liu X., Hu Y., Ming Y., Bai M., Liu J., Xiao K., Zeng Q., Yang J. (2023). Whole Genome Sequencing Reveals Autooctoploidy in the Chinese Sturgeon and its Evolutionary Trajectories. Genom. Proteom. Bioinform..

[33] Cheng X., Xiao K., Jiang W., Peng G., Chen P., Shu T., Huang H., Shi X., Yang J. (2024). Selection, identification and evaluation of optimal reference genes in Chinese sturgeon (*Acipenser sinensis*) under polypropylene microplastics stress. Sci. Total Environ..

[34] Livak K.J., Schmittgen T.D. (2001). Analysis of relative gene expression data using real-time quantitative PCR and the 2(-Delta Delta C(T)) Method. Methods.

[35] Hallgren J., Tsirigos K., Pedersen M.D., Almagro Armenteros J.J., Marcatili P., Nielsen H., Krogh A., Winther O. (2022). DeepTMHMM predicts alpha and beta transmembrane proteins using deep neural networks. BioRxiv.

[36] Thompson J.D., Higgins D.G., Gibson T.J. (1994). CLUSTAL W: Improving the sensitivity of progressive multiple sequence alignment through sequence weighting, position-specific gap penalties and weight matrix choice. Nucleic Acids Res..

[37] Hall T. (1999). *BioEdit*: A user-friendly biological sequence alignment editor and analysis program for Windows 95/98/NT. Nucleic Acids Symp. Ser..

[38] Abramson J., Adler J., Dunger J., Evans R., Green T., Pritzel A., Ronneberger O., Willmore L., Ballard A.J., Bambrick J. (2024). Accurate structure prediction of biomolecular interactions with AlphaFold 3. Nature.

[39] Li M., Tang H., Qing R., Wang Y., Liu J., Wang R., Lyu S., Ma L., Xu P., Zhang S. (2024). Design of a water-soluble transmembrane receptor kinase with intact molecular function by QTY code. Nat. Commun..

[40] Tamura K., Stecher G., Kumar S. (2021). MEGA11: Molecular Evolutionary Genetics Analysis Version 11. Mol. Biol. Evol..

[41] Zheng X., Ding Z., Xu Y., Monroig O., Morais S., Tocher D.R. (2009). Physiological roles of fatty acyl desaturases and elongases in marine fish: Characterisation of cDNAs of fatty acyl Δ6 desaturase and *elovl5* elongase of cobia (*Rachycentron canadum*). Aquaculture.

[42] Hastings N., Agaba M., Tocher D.R., Leaver M.J., Dick J.R., Sargent J.R., Teale A.J. (2001). A vertebrate fatty acid desaturase with Delta 5 and Delta 6 activities. Proc. Natl. Acad. Sci. USA.

[43] Christie W.W. (1998). Gas chromatography-mass spectrometry methods for structural analysis of fatty acids. Lipids.

[44] Monroig Ó., Lopes-Marques M., Navarro J.C., Hontoria F., Ruivo R., Santos M.M., Venkatesh B., Tocher D.R., Castro L.F.C. (2016). Evolutionary functional elaboration of the *Elovl2/5* gene family in chordates. Sci. Rep..

[45] Jaillon O., Aury J.-M., Brunet F., Petit J.-L., Stange-Thomann N., Mauceli E., Bouneau L., Fischer C., Ozouf-Costaz C., Bernot A. (2004). Genome duplication in the teleost fish *Tetraodon nigroviridis* reveals the early vertebrate proto-karyotype. Nature.

[46] Castro L.F.C., Monroig Ó., Leaver M.J., Wilson J., Cunha I., Tocher D.R. (2012). Functional Desaturase *Fads1* (Δ5) and *Fads2* (Δ6) Orthologues Evolved before the Origin of Jawed Vertebrates. PLoS ONE.

[47] Glasauer S.M.K., Neuhauss S.C.F. (2014). Whole-genome duplication in teleost fishes and its evolutionary consequences. Mol. Genet. Genom..

[48] Tocher D.R. (2003). Metabolism and Functions of Lipids and Fatty Acids in Teleost Fish. Rev. Fish. Sci..

[49] Xie D., Fu Z., Wang S., You C., Monroig Ó., Tocher D.R., Li Y. (2018). Characteristics of the *Fads2* gene promoter in marine teleost *epinephelus coioides* and role of SP1-binding site in determining promoter activity. Sci. Rep..

[50] Gillard G., Harvey T.N., Gjuvsland A., Jin Y., Thomassen M., Lien S., Leaver M., Torgersen J.S., Hvidsten T.R., Vik J.O. (2018). Life-stage-associated remodelling of lipid metabolism regulation in Atlantic salmon. Mol. Ecol..

[51] Mourente G., Rodríguez A., Grau A., Pastor E. (1999). Utilization of lipids by *Dentex dentex* L. (Osteichthyes, Sparidae) larvae during lecitotrophia and subsequent starvation. Fish Physiol. Biochem..

[52] Sargent J.R., Tocher D.R., Bell J.G., Halver J.E., Hardy R.W. (2003). 4—The Lipids. Fish Nutrition.

[53] Monroig O., Rotllant J., Sánchez E., Cerdá-Reverter J.M., Tocher D.R. (2009). Expression of long-chain polyunsaturated fatty acid (LC-PUFA) biosynthesis genes during zebrafish *Danio rerio* early embryogenesis. Biochim. Biophys. Acta.

[54] You C., Miao S., Lin S., Wang S., Waiho K., Li Y. (2017). Expression of long-chain polyunsaturated fatty acids (LC-PUFA) biosynthesis genes and utilization of fatty acids during early development in rabbitfish *Siganus canaliculatus*. Aquaculture.

[55] Tocher D.R., Leaver M.J., Hodgson P.A. (1998). Recent advances in the biochemistry and molecular biology of fatty acyl desaturases. Prog. Lipid Res..

[56] Datsomor A.K., Zic N., Li K., Olsen R.E., Jin Y., Vik J.O., Edvardsen R.B., Grammes F., Wargelius A., Winge P. (2019). CRISPR/Cas9-mediated ablation of *elovl2* in Atlantic salmon (*Salmo salar* L.) inhibits elongation of polyunsaturated fatty acids and induces *Srebp-1* and target genes. Sci. Rep..

[57] Xie D., Wang S., You C., Chen F., Tocher D.R., Li Y. (2015). Characteristics of LC-PUFA biosynthesis in marine herbivorous teleost *Siganus canaliculatus* under different ambient salinities. Aquac. Nutr..

[58] Li Y., Hu C., Zheng Y., Xia X., Xu W., Wang S., Chen W., Sun Z., Huang J. (2008). The effects of dietary fatty acids on liver fatty acid composition and Δ6-desaturase expression differ with ambient salinities in *Siganus canaliculatus*. Comp. Biochem. Physiol. B Biochem. Mol. Biol..

[59] Nayak M., Giri S.S., Pradhan A., Samanta M., Saha A. (2020). Effects of dietary α-linolenic acid/linoleic acid ratio on growth performance, tissue fatty acid profile, serum metabolites and Δ6 *fad* and *elovl5* gene expression in silver barb (*Puntius gonionotus*). J. Sci. Food Agric..

[60] Datsomor A.K., Gillard G., Jin Y., Olsen R.E., Sandve S.R. (2022). Molecular Regulation of Biosynthesis of Long Chain Polyunsaturated Fatty Acids in Atlantic Salmon. Mar. Biotechnol..

[61] Asif M. (2011). Health effects of omega-3,6,9 fatty acids: *Perilla frutescens* is a good example of plant oils. Orient. Pharm. Exp. Med..

[62] Orsavova J., Misurcova L., Ambrozova J.V., Vicha R., Mlcek J. (2015). Fatty Acids Composition of Vegetable Oils and Its Contribution to Dietary Energy Intake and Dependence of Cardiovascular Mortality on Dietary Intake of Fatty Acids. Int. J. Mol. Sci..

[63] Tocher D.R., Betancor M.B., Sprague M., Olsen R.E., Napier J.A. (2019). Omega-3 Long-Chain Polyunsaturated Fatty Acids, EPA and DHA: Bridging the Gap between Supply and Demand. Nutrients.

