# Supplementary material for: Molecular Identification and Functional Characterization of LC-PUFA Biosynthesis Elongase (elovl2) Gene in Chinese Sturgeon (Acipenser sinensis)
Source: Animals (Basel). 2024 Aug 14;14(16):2343. doi: 10.3390/ani14162343 (PMC11350804; doi:10.3390/ani14162343)
Supplement: Supplementary file 1 [file animals-14-02343-s001.zip › animals-3087388-supplementary.pdf]

Table S1. Formulation and proximate composition of the experimental diets.

	Experimental diets	
	**VO**	**FO**
Ingredients(%)		
Fishmeal[a]	20.00	20.00
Fermented soybean meal[b]	15.00	15.00
Soya concentrated[c]	25.00	25.00
Corn protein concentrate	10.00	10.00
Mixed vegetable oil[d]	7.57	-
Fish oil	-	7.57
Starch	16.63	16.63
Mineral mix[e]	2.00	2.00
Vitamin mix[f]	2.00	2.00
Dicalcium phosphate	1.00	1.00
Choline	0.50	0.50
Betaine	0.30	0.30
Proximate composition (%)		
Moisture	11.76	11.42
Crude protein (dry mass)	50.46	50.65
Crude lipid (dry mass)	9.37	9.42
Ash (dry mass)	11.63	11.63

[a] Fishmeal: 72% Crude protein; 8.75% Crude lipid (DHA 8.5%, EPA 0.4%).

[b] Fermented soybean meal: 57% Crude protein; 3.2% Crude lipid.

[c] Soya concentrated: 85% Crude protein; 0.8% Crude lipid.

[d] Mixed vegetable oil: Perilla oil/Palm oil/Soybean oil=1:1:1.

[e] Mineral mix: Fe citrate, 8000~48,000mg; $CuSO_4$, 1000~3000mg; $ZnSO_4$, 4000~12,000mg; $MnSO_4$, 2500 ~ 7500mg; $MgSO_4$, 24,000 ~ 72,000mg; Moisture ≤ 10%.

[f] Vitamin mix: Vitamin A; 400,000 ~ 1200,000 IU; Vitamin D_3, 200,000 ~ 600,000 IU; Vitamin B_1 ≥ 1200mg; Vitamin B_6 ≥ 3000mg; Vitamin B_{12} ≥ 3000ug; Potassium ≥ 10,000 mg; Sodium, 60,000 ~ 180,000 mg; Moisture ≤ 10%.

Table S2. Nucleotide sequences of the primers used for PCR.

Target genes	Primer sequences (5′-3′)
**Primers for full-length cDNA cloning**	
elovl2-F	GCAGCCCAGCCTACAGACCA
elovl2-R	TGCCGCTCCCTCCTCACTT
**Primers for cDNA ORF cloning**	
elovl2-F- KpnI	GGGGTACCATGGGAGCCGGTTCGGATGA
elovl2-R- EcoRI	CGGAATTCTCACTTGTGCAGGTACGCGT
**Primers for real-time quantitative PCR (qPCR)**	
QS-*elovl2*	CGCTGCTGTTGTCTCTTGT
QA-*elovl2*	CATTGTGCCATTAGTTGTTGCT
QS-18s	CGCCGAGAAGACGATCAAAC
QA-18s	TGATCCTTCCGCAGGTTCAC

Table S3. *elovls* IDs used for phylogenetic analysis. Data were collected from Ensembl or GenBank.

Number	Species	Gene name	Gene ID
1	*Homo sapiens*	*ELOVL2*	NP_060240.3
2	*Anolis carolinensis*	*elovl2*	XP_008113195.1
3	*Callorhinchus milii*	*elovl2*	XP_007900820.1
4	*Acipenser ruthenus*	*elovl2*	XP_033856140.1
5	*Danio rerio*	*elovl2*	NP_001035452.1
6	*Salmo salar*	*elovl2*	NP_001130025.1
7	*Homo sapiens*	*ELOVL5*	NP_001288785.1
6	*Anolis carolinensis*	*elovl5*	XP_062839043.1
7	*Callorhinchus milii*	*elovl5*	XP_042188641.1
8	*Acipenser ruthenus*	*elovl5*	XP_058881019.1
9	*Danio rerio*	*elovl5*	NP_956747.1

10	*Salmo salar*	*elovl5*	NP_001130024.1
11	*Homo sapiens*	*ELOVL4*	NP_073563.1
12	*Anolis carolinensis*	*elovl4*	XP_003215742.1
13	*Callorhinchus milii*	*elovl4a*	XP_042189385.1
14	*Acipenser ruthenus*	*elovl4a*	XP_058880593.1
15	*Danio rerio*	*elovl4b*	NP_956266.1
16	*Salmo salar*	*elovl4b*	XP_014060878.1
17	*Lepisosteus oculatus*	*elovl8a*	XP_015210847.1
18	*Danio rerio*	*elovl8a*	NP_001070061.1
19	*Salmo salar*	*elovl8a*	XP_013995966.1
20	*Danio rerio*	*elovl8b*	NP_001191453.1
21	*Salmo salar*	*elovl8b*	XP_014071374.2
22	*Homo sapiens*	*ELOVL6*	NP_001124193.1
23	*Anolis carolinensis*	*elovl6*	XP_008110313.1
24	*Callorhinchus milii*	*elovl6*	AFO95801.1
25	*Danio rerio*	*elovl6*	NP_955826.1
26	*Salmo salar*	*elovl6*	XP_014054664.1

Table S4. Sequence identity calculation of Elovl2s among different species

**Seq Identity**	**Hs_Elovl2**	**Ac_Elovl2**	**Xt_Elovl2**	**Cm_Elovl2**	**As_Elovl2**	**Dr_Elovl2**
Hs_Elovl2	ID	76.82	74.83	72.19	73.51	65.23
Ac_Elovl2	76.82	ID	78.15	74.17	75.17	66.56
Rt_Elovl2	74.83	78.15	ID	75.17	75.17	67.22
Cm_Elovl2	72.19	74.17	75.17	ID	73.18	65.89
**As_Elovl2**	**73.51**	**75.17**	**75.17**	**73.18**	**ID**	**70.20**
Dr_Elovl2	65.23	66.56	67.22	65.89	70.20	ID

Species used in present study include human (*Homo sapiens*; Hs), green anole (*Anolis carolinensis*; Ac), Common frog (*Rana temporaria*; Rt), elephant shark (*Callorhinchus milii*; Cm), Chinese sturgeon (*Acipenser sinensis*; As), and zebrafish (*Danio rerio*; Dr).
